# Philanthro-metrics: Mining multi-million-dollar gifts

**DOI:** 10.1371/journal.pone.0176738

**Published:** 2017-05-26

**Authors:** Una O. Osili, Jacqueline Ackerman, Chin Hua Kong, Robert P. Light, Katy Börner

**Affiliations:** 1 Indiana University Lilly Family School of Philanthropy, Indianapolis, Indiana, United States of America; 2 Cyberinfrastructure for Network Science Center, School of Informatics and Computing, Indiana University, Bloomington, Indiana, United States of America; 3 Indiana University Network Science Institute, Bloomington, Indiana, United States of America; Iowa State University, UNITED STATES

## Abstract

The Million Dollar List (MDL, online at http://www.milliondollarlist.org) is a compilation of publicly announced charitable donations of $1 million or more from across the United States since 2000; as of December 2016, the database contains close to 80,000 gifts made by U.S. individuals, corporations, foundations, and other grant-making nonprofit organizations. This paper discusses the unique value of the Million Dollar List and provides unique insights to key questions such as: How does distance affect giving? How do networks impact million-dollar-plus gifts? Understanding the geospatial and temporal dimensions of philanthropy can assist researchers and policymakers to better understand the role of private funding in innovation and discovery. Moreover, the results from the paper emphasize the importance of philanthropy for fueling research and development in science, the arts, environment, and health. The paper also includes the limitations of the presented analyses and promising future work.

## Introduction

As public funding for research declines, scientists are increasingly seeking private donors to support research and discovery [1]. In fact, private philanthropy is a growing share of funding for scientific research with donations from U.S. foundations to science, technology, and medical research playing an important role [2].

Philanthropy—particularly large gifts—can play a key role in fueling scientific advances, or serving as a catalyst for research in new areas in health, education, environment, and other key sectors. Philanthropic support also fuels innovation in how services are delivered and can raise awareness around local and global health issues such as HIV/AIDS and malaria.

In this paper, we use data from the Million Dollar List and apply temporal, geospatial, and topical analyses to understand the dynamics of U.S. philanthropy, the geospatial and topical distributions of donors and recipients, as well as the networks that interconnect them. Researchers and policymakers have focused an increasing amount of attention on the potential for private funding to solve problems and address urgent needs in society. Despite significant attention, there have been very few studies that examine the geographic dispersion of million dollar gifts and how this changes over time, due in part to data limitations.

The Million Dollar List (MDL) is the most comprehensive and up-to-date record of gifts of $1 million and more in the U.S. It has been compiled and studied at the Indiana University Lilly Family School of Philanthropy since 2000, and a number of published and working papers have resulted. One study [3] explored the effects of key economic indicators on million-dollar giving, and demonstrated that negative economic conditions results in fewer million-dollar-plus gifts from individuals, but have a counter-cyclical effect on foundation giving, which increases during times of recession. MDL data was also used to understand the role of philanthropy in response to government cutbacks and to identify approaches to partnerships between private philanthropy and the government; the study found that 40 percent of million-dollar-plus gifts in the sample were connected to government in some way, including to publicly funded higher education institutions [4]. Million-dollar gifts to higher education were analyzed to determine the aspects of institutions more likely to receive greater numbers of gifts; the study identified a number of indicators that were related to such giving, including school ranking, tenured faculty, and endowment value [5]. The data was also used to analyze which companies or corporate foundations give overseas, and to what areas; the study found that companies were more likely to give overseas if they had at least one foreign subsidiary, and if they had a larger share of overseas sales revenue [6]. Working papers currently underway also explore MDL data, including topics such as the impact of community characteristics on the million-dollar gifts received by a community [7], and flows of private aid for international development [8].

A number of other data sources shed light on a variety of aspects of giving, including information from the Foundation Center [9] on giving by foundations, and the *Chronicle of Philanthropy’s* data [10] on top donors and recipients. The MDL is unique in its focus on the top strata of gifts worth at least $1 million, as well as its gift-level data collection (as opposed to looking at aggregate amounts donated or received).

This paper proceeds with a description of the MDL data. We then demonstrate three exemplary analyses and visualizations of the data. All workflows are documented in detail and all data used here is publicly available for future studies. The Scaling Philanthropy: Providing New Insight About Million Dollar Gifts project was funded by the Bill & Melinda Gates Foundation. The funders had no role in study design, data collection and analysis, decision to publish, or preparation of the manuscript. The Scaling Philanthropy project was designed to support data collection and an in-depth validation study on gifts of a million dollars and above in the U.S. by donor and recipient type over time. Despite significant attention, there have been very few studies that examine million dollar gifts, and the objective of the project was to expand data and knowledge on large gifts.

## Materials and methods

The MDL is a database that compiles charitable donations worth at least $1 million (USD) given by donors in the U.S. to various causes; the database is available on a dedicated website (http://www.milliondollarlist.org) and has been compiled since 2000 (data used for these analyses can be found at https://figshare.com/articles/Million_Dollar_List_full_data_2000-2012_used_for_PLOS_ONE_paper/4970558). The data include gifts made by individuals, corporations, foundations and other grant-making nonprofit organizations. Data is compiled using two methods. First, qualifying gifts are identified through web crawlers and other online resources, such as publications of the *Chronicle of Philanthropy* and the *Chronicle of Higher Education*, the *Philanthropy News Digest* from the Foundation Center, and email alerts from a number of web searches. Second, additional data based on information from tax returns is purchased from FoundationSearch, which enables the MDL to encompass a wider range of data, as many foundation gifts are not publicly reported.

Once qualifying gifts are identified, researchers code each gift and enter it into a central database. Information collected includes donor name, city, state, and type; recipient name, city, state, country, and subsector; gift amount and notes, source of information, date reported, and year and quarter of the donation.

While the MDL remains the most complete picture of million-dollar giving in the U.S., it has its limitations. For example, purchased data from FoundationSearch is included in the MDL database with a two to three year delay. FoundationSearch data has improved its coverage and accuracy in recent years due to continual improvements in data collection methods, and thus is not appropriate for inclusion in time-series analysis. For these reasons, much analysis, particularly involving trends over time, has been conducted while omitting FoundationSearch data, which comprises approximately two-thirds of the dataset. This may be more accurate in terms of trends over time, but it also means that foundation gifts are under-reported, since foundations are less likely to seek publicity for all of their million-dollar-plus gifts.

An important concern with the MDL data is that due to the collection methodology, certain gifts may be over- or under-reported. First, religious organizations and small nonprofits are less likely to publicly report or obtain media coverage of high-dollar gifts. Second, public reports of certain gifts may differ from the actual value of the gift, for example in-kind contributions or non-monetary gifts, such as stocks. Finally, there may be some duplicate reporting due to the timing of media reporting on contributions; this is not expected to have a major impact on the MDL as extensive efforts have been undertaken to remove duplicate gifts. Due in part to the challenges mentioned above, validation of the data and methodology was undertaken and completed in 2014. The validation involved several steps. First, million-dollar gifts from foundations and corporate foundations were validated by comparing tax data from FoundationSearch with data from public announcements. Second, a survey was conducted of nonprofits in four U.S. cities to determine the accuracy and extent of public announcements of million-dollar gifts from all sources, including individuals.

The dataset used in this study can be found at http://www.milliondollarlist.org/data. It includes public announcements from the 2000–2014 calendar years, and FoundationSearch (tax) data from the 2000–2011 calendar years. It comprises 76,761 gifts given to 18,211 unique recipients in 124 countries (55 recipients have no country information) totaling $440 billion. The gifts were made from 2000 to 2014 by 14,937 donors (plus 1,032 donations from anonymous donors), all from the United States. The donors were classified as corporations (1,519), foundations (6,531), or individuals (6,887). 52,247 of the records (68.06 percent of the total dataset) were retrieved via FoundationSearch. There are different charitable subsectors, specifically: *Arts*, *Culture*, *and Humanities; Education (Non-Higher Education); Environment and Animals; Foundation; Government; Health; Higher Education; Human Services; International; Overseas; Public*, *Society Benefit; Religious Organizations*; and *Various*. Please note that for some of the analyses below, gifts to the *Foundation* or *Various* subsectors were excluded. This is because some gifts to foundations are at risk for double-counting in the MDL, because typically these gifts are then granted out from foundations to front-line nonprofits; and because gifts classified as *Various* go to many different types of organizations.

## Results

Three different analyses were conducted to answer long-standing questions about the dynamics of U.S. philanthropy, the geospatial and topical distributions of donors and recipients, as well as the networks that interconnect them. The dynamic and spatial dimensions of philanthropy provide insights that can improve the understanding of how philanthropy can contribute to funding scientific innovation and discovery and how this changes over time, a previously underexplored subject. Details on workflows, results, and interpretations are provided in references for each analysis.

### Local vs. Global giving

Our first analysis aims to explore whether U.S. donors give more locally or globally. This requires the identification of donor and recipient geolocations, computing great circle distances for each donor and recipient pair, and plotting results so that patterns and trends can be identified and communicated.

In the data, donor geolocations are missing for six gifts and recipient geolocations are unavailable for 118 gifts. The missing recipients include 55 that simply have no data. All other donors and recipients were geolocated based on their combined city, state, and country information using the Bing! geocoder available in the Sci2 Tool [11], detailed instructions are available [12]. Exactly 76,744 donors (99.99 percent) and 76,643 recipients (99.84 percent) could be geolocated. Of the 439.8 billion dollars in donations, 99.99 percent were geolocated by donor and 99.77 percent by recipient.

**Fig 1:**
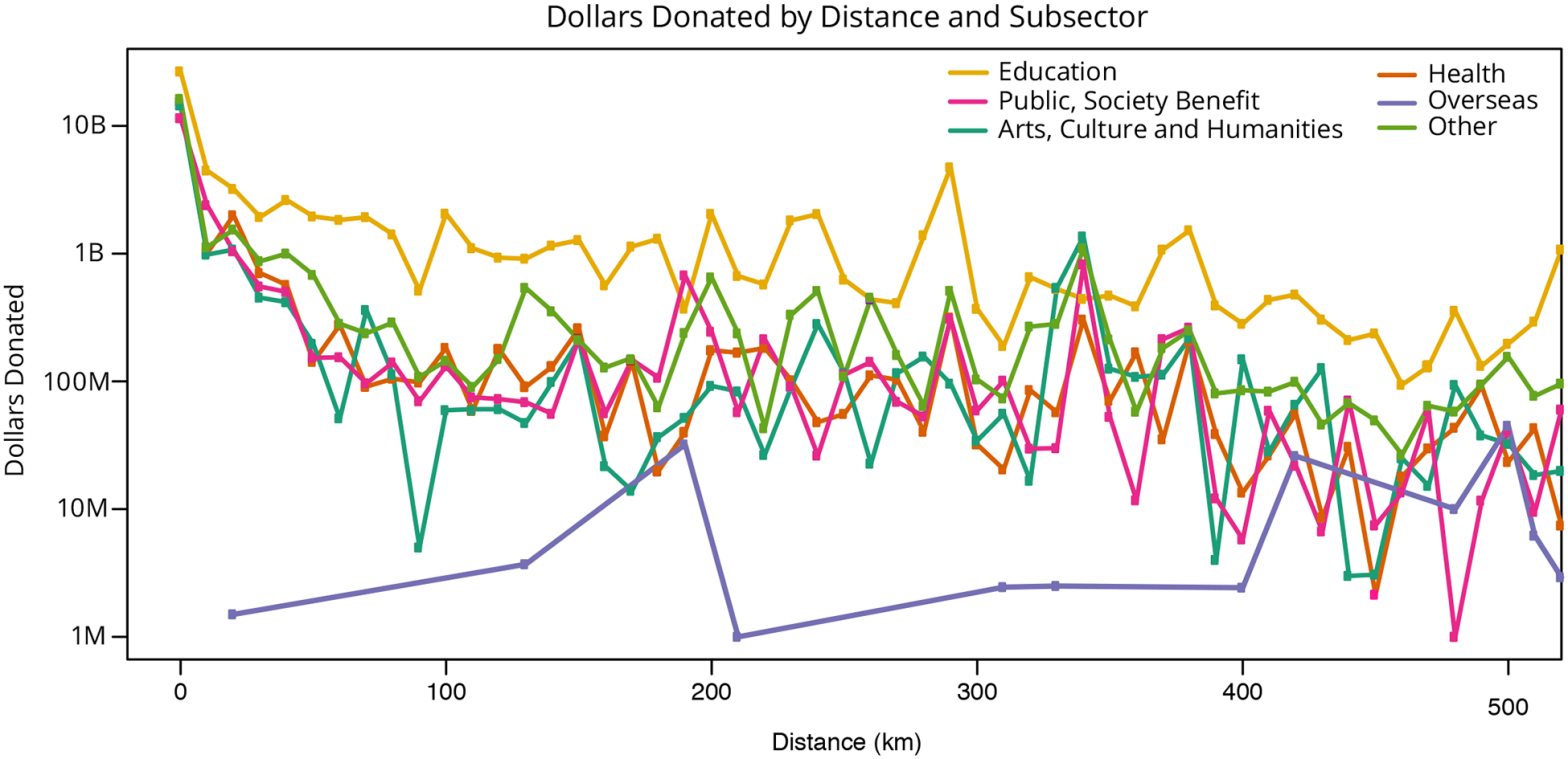
Dollars of million-dollar-plus gifts (2000–2014) over distance for major subsectors rounded to the nearest 10 km as a form of binning.

**Fig 2:**
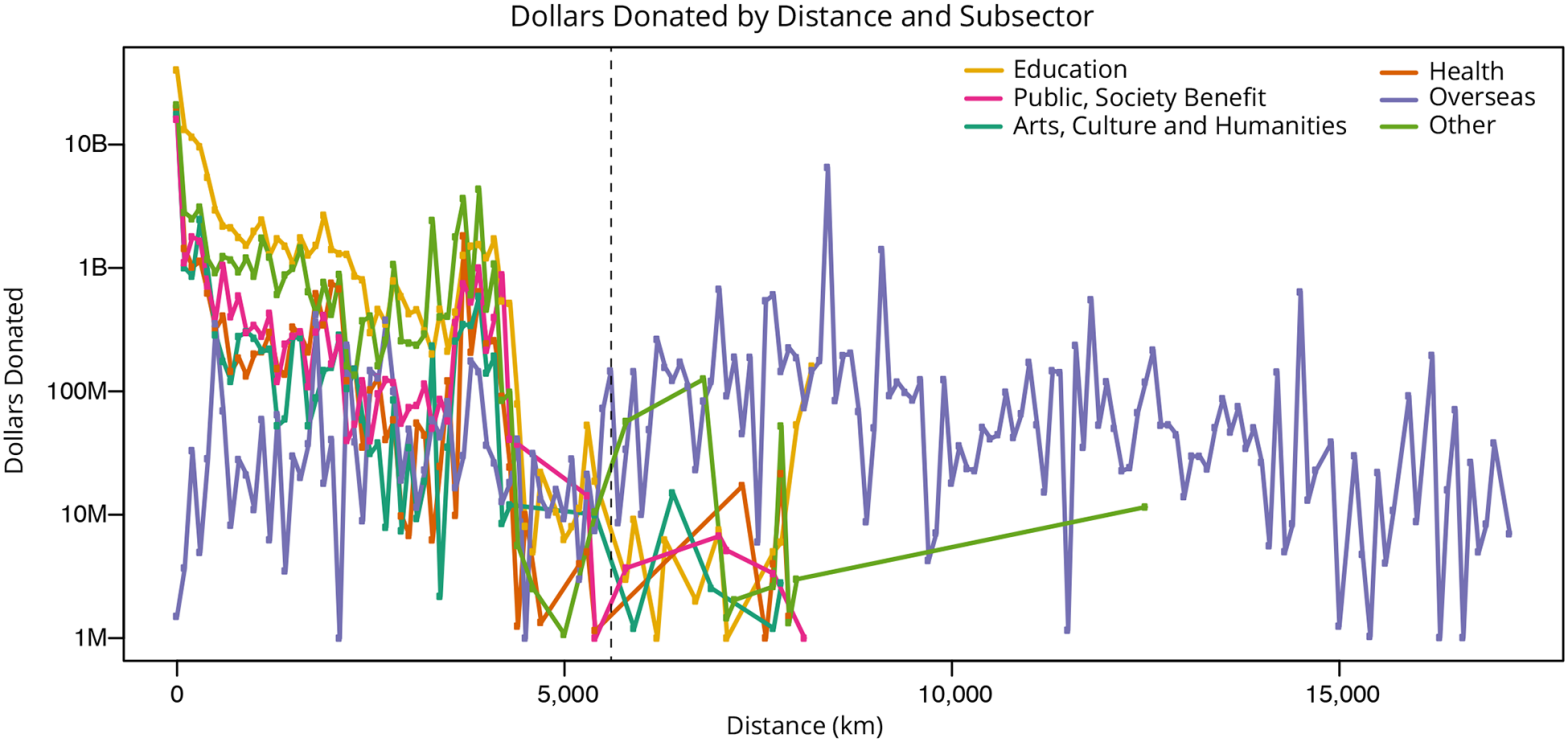
Dollars of million-dollar-plus gifts (2000–2014) over distance for major subsectors rounded to the nearest 100 km as a form of binning.

Next, the great circle distances were calculated for all geolocated donor-recipient pairs. The minimum distance is zero, indicating that the donor and recipient are in the very same geolocation, the maximum distance is 17,182 km (compared to a maximum possible distance of 20,015 km—half the circumference of Earth). Figs 1 and 2 plot the number of dollars over distance for all subsectors, (excluding the *Foundation* and *Various* subsectors for reasons mentioned above) with distance rounded to the nearest 10km (in Fig 1) and 100 km (in Fig 2) as a form of binning. As can be seen, *Education (Non-Higher Education)* gifts (yellow line) are mostly given locally, while the *Overseas* gifts (purple line) have the greatest distance between donors and recipients. In general, donors tend to give closer to home; see zoom into 0 to 520 km distance in Fig 1.

**Fig 3:**
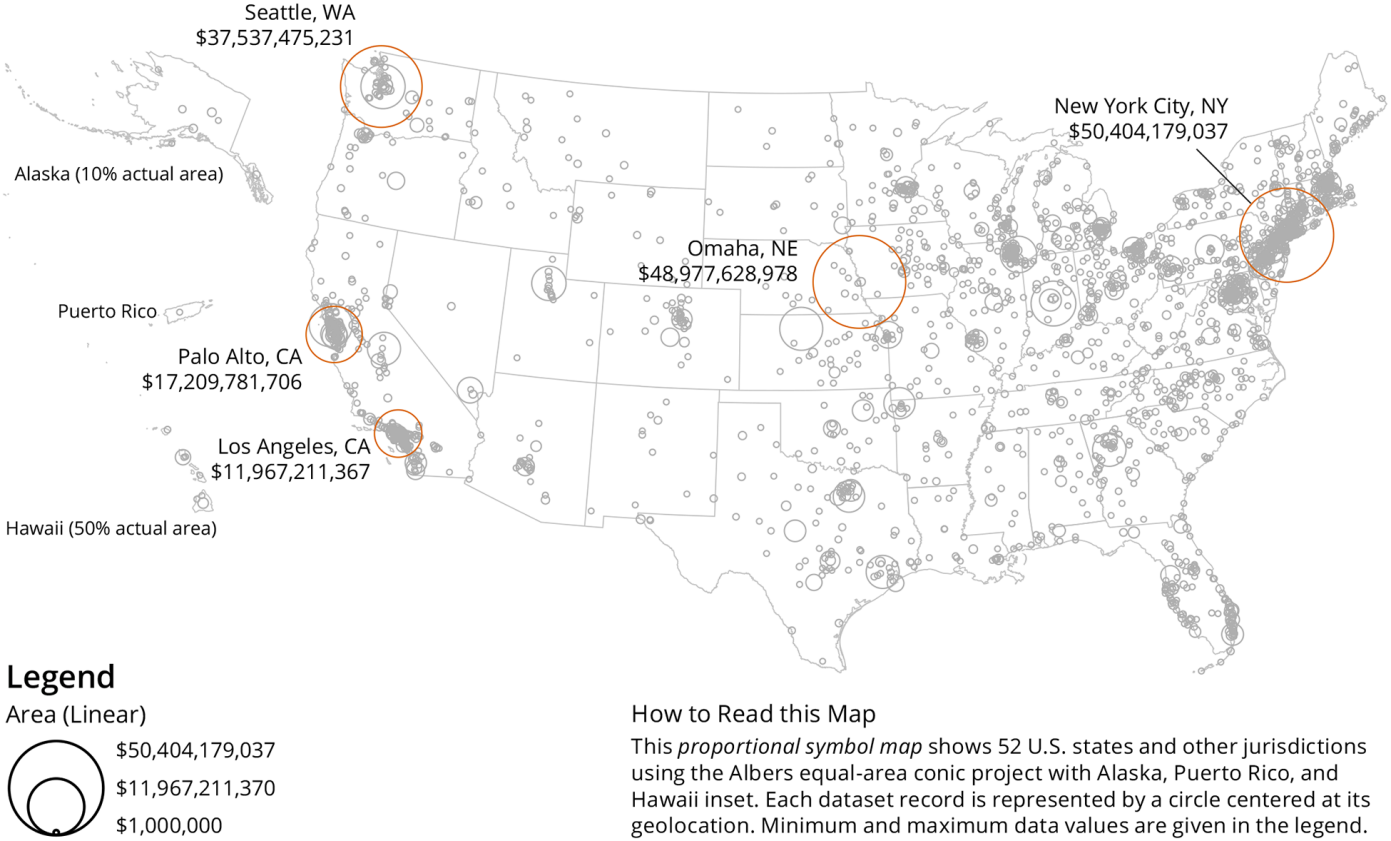
U.S. map with geolocations of donors of million-dollar-plus gifts, 2000–2014. The top-five donors’ geolocations are labelled and the total dollar value is given.

**Fig 4:**
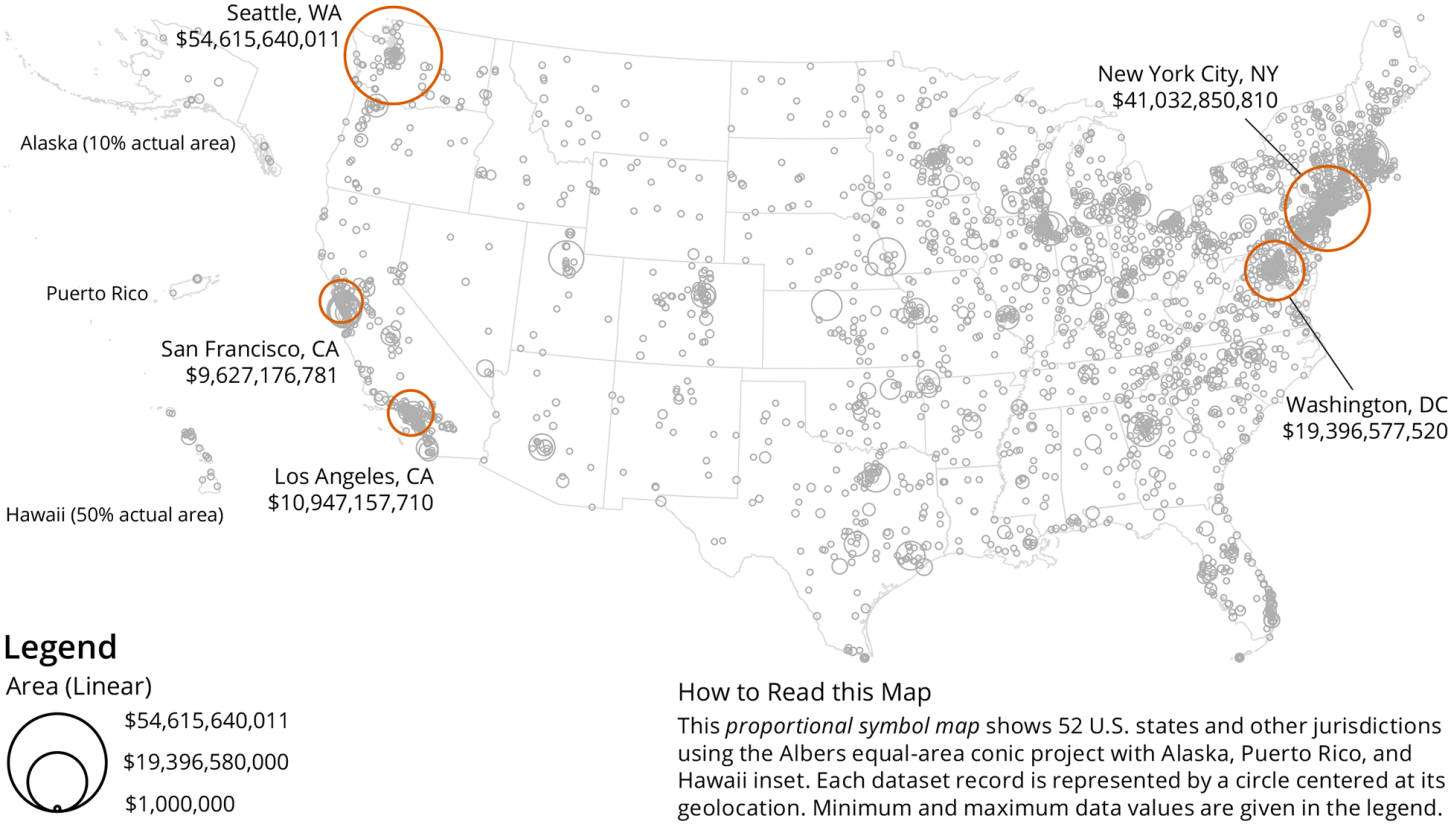
U.S. map with geolocations of recipients of million-dollar-plus gifts, 2000–2014. The top-five recipients’ geolocations are labelled and the total dollar value is given.

**Fig 5:**
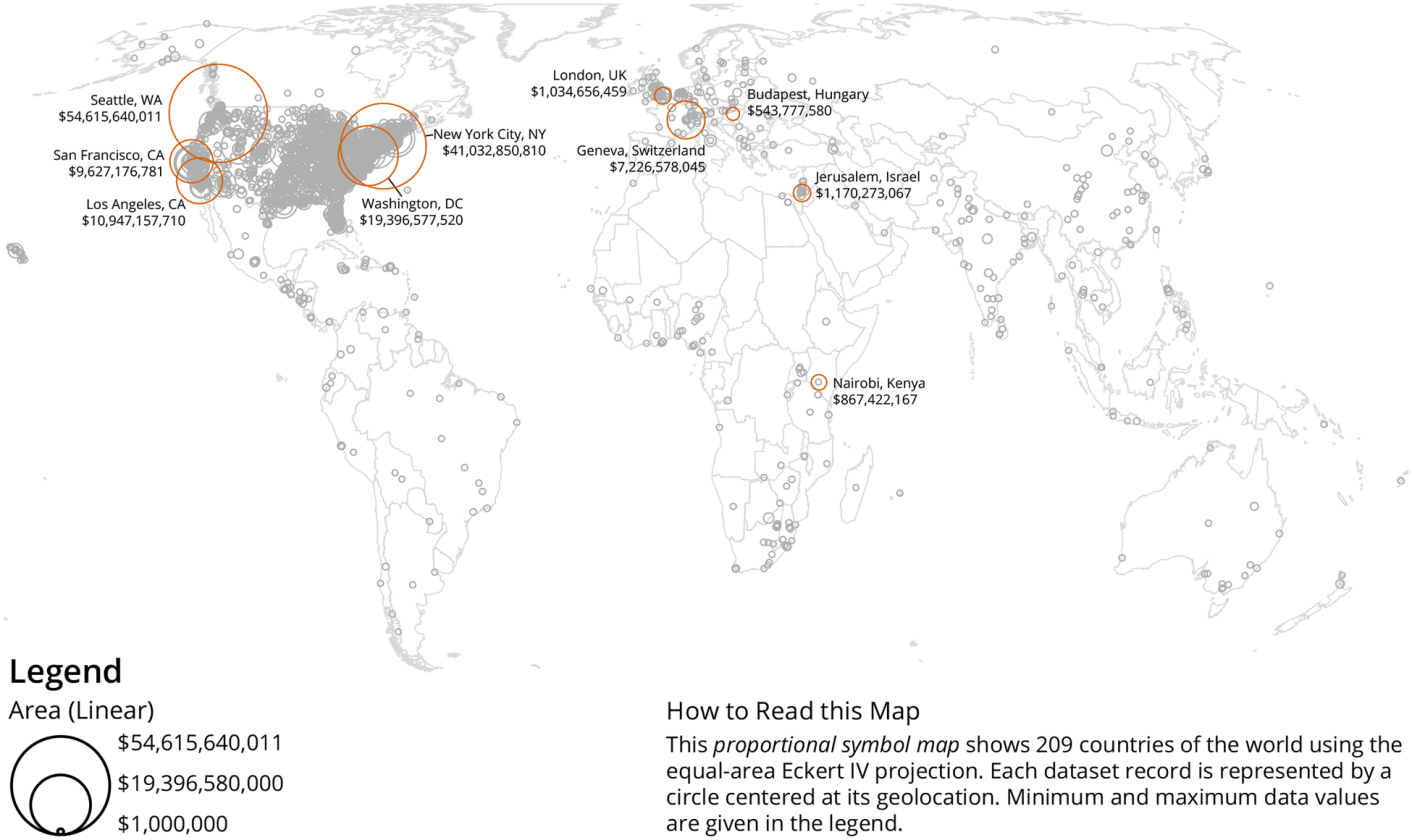
World map with geolocations of recipients of million-dollar-plus gifts, 2000–2014. The top-ten recipients’ geolocations are labelled and the total dollar value is given.

An important objective was to understand the geospatial distribution of donors and recipients and proportional symbol maps were created for both using the Sci2 Tool [12]. As the dataset is U.S. centric, a U.S. map was used as a basemap. Data overlays of donors are shown in Fig 3 and recipients are depicted in Fig 4. In both maps, the area of each circle is proportional to the total value of donations per unique geolocation. As can be seen, the vast majority of gifts are being given to recipient organizations in the U.S., with heavy clusters in major cities like Washington, DC and New York City, NY. We also see clusters in cities where major donors are located, such as Seattle, WA, the home of the Bill & Melinda Gates Foundation. A world map with geolocations of recipients is given in Fig 5. For the global map, while the U.S. receives the bulk of the gifts, there are also concentrations of gifts in Europe, especially the United Kingdom, Israel, and Switzerland. This affirms the point made above, that philanthropy in the U.S. tends to be local; when looking at all gifts together, we see that gifts tend to stay within the U.S.

### Networks of giving

We also examined which donors give to which subsectors, the data was prepared to extract a bi-modal network of “donor name” to “recipient subsector” for the 33 donors who gave $1 billion or more in 2000 to 2014; see Table 1 for a listing of the number of gifts. The 13 subsectors were collapsed into five: “Education” (combining *Education (Non-Higher Education)* with *Higher Education*); “International” (combining *International* with *Overseas*); “Health;” “Foundations;” and “Other” (combining all remaining subsectors, except *Various*, which was excluded from this analysis for reasons mentioned above). Foundations are included in this analysis because these top 33 donors gave disproportionately to that subsector, and to exclude these gifts would provide an unclear picture of the data.

**Table 1:** Number of gifts by 33 billion-dollar donors to four collapsed subsectors.

**All Education**	**6.089**
Education (Non-Higher Education)	1,756
Higher Education	4,333
**International/Overseas**	**3,315**
International	1,595
Overseas	1,720
**Health**	**976**
**Other**	**6,454**
Arts, Culture, and Humanities	1,188
Environment and Animals	742
Government	127
Human Services	2,012
Public, Society Benefit	2,284
Religious Organizations	101
**Foundations**	**190**
**Various** (excluded)	**35**

**Fig 6:**
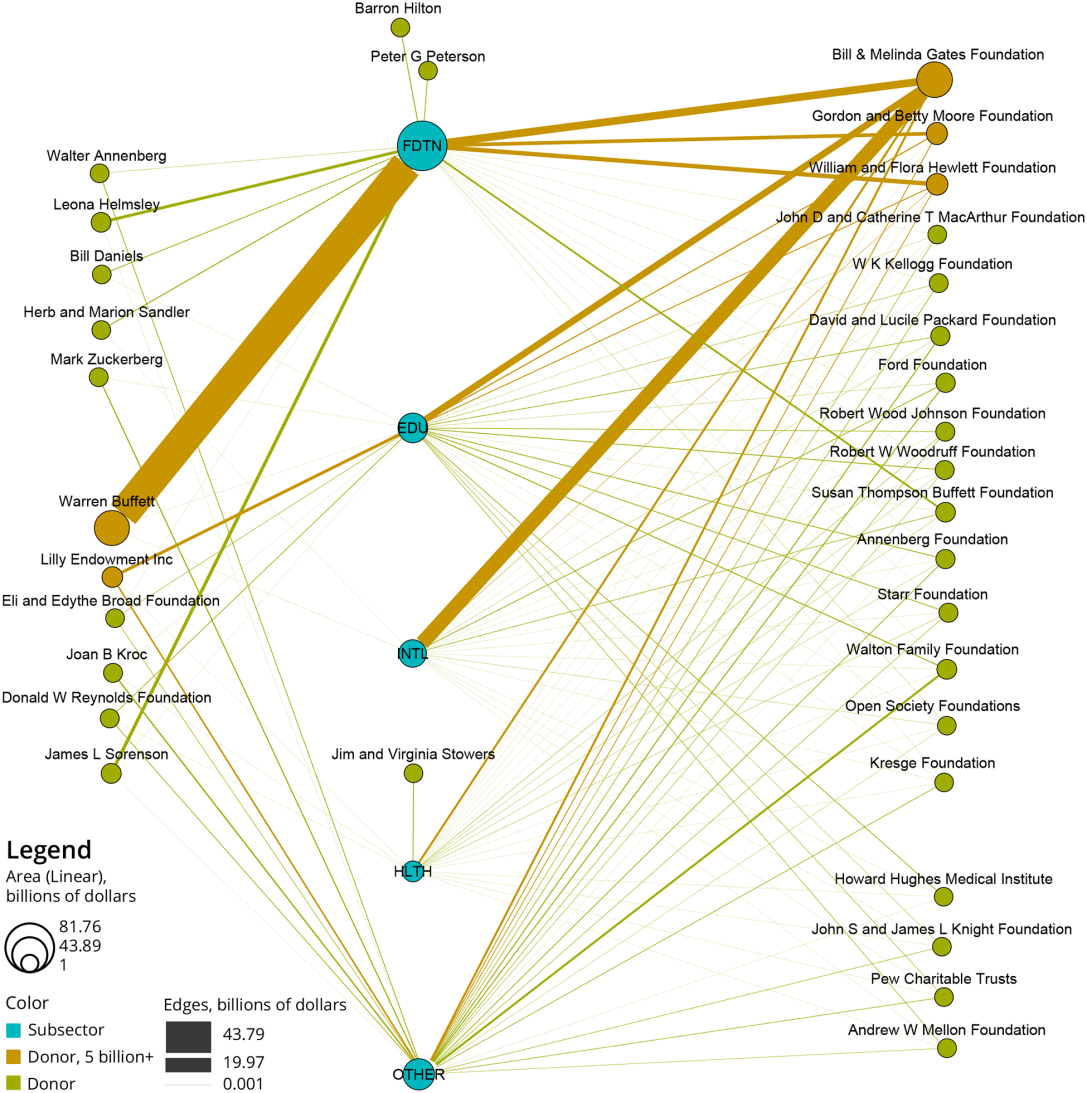
Bi-modal network of “donor name” to merged subsectors for the 33 billion-dollar donors that gave to subsectors from 2000 to 2014. Node size and line thickness denote total dollar value donated.

Next, a bi-modal network of the 33 donors and the four merged subsectors was extracted using the Sci2 Tool, and detailed instructions are available [13]. The network has two node types: donors and subsectors, see Fig 6 and legend. All subsector nodes were placed on a vertical axis on the middle of the figure. Donors were grouped by the number of subsectors they fund and according to their funding profiles (e.g., donors that give exclusively to one subsector are placed close to that respective node). Donor and subsector nodes are area sized by total dollar value. The edges are weighted and thickness coded by total dollar value given. For example, the Bill and Melinda Gates Foundation (in orange, top-right) gives primarily to the “International” and “Foundations” subsectors (3,566 gifts), but they also give to the “Education” subsector, and less to the remaining two subsectors. Warren Buffet (orange, left middle) gives mostly to “Foundations,” with some smaller donations to “Health” and “International.” The Lilly Endowment Inc. (in orange, on left) gives a much smaller number of larger gifts, totaling more than $5 billion, to three subsectors in a more balanced way. We note that only certain combinations of subsectors exist, and only three donors support exactly one subsector. While co-funding between multiple major donors is taking place for certain subsectors, other causes have fewer funders giving exclusively to those issues.

## Discussion

At the present time, the Million Dollar List dataset, which is publicly available, has incredible transformative potential for the philanthropic sector, along with many other areas of study. The MDL data can be used to answer a wide range of questions related to philanthropic giving, including:

- Which types of corporations tend to give at the million-dollar-plus level, and to which charities or charitable subsectors?
- Are there gender differences in million-dollar-plus giving?
- How have gifts to current issues (such as climate change, income inequality, etc.) changed over time?
- How does private philanthropy compare with other funding sources (such as government funding, development assistance, social entrepreneurship, social investing, etc.) for various target areas?
- Do changes in the tax code impact the number and total amount of million-dollar-plus gifts given?

The Million Dollar List is an invaluable resource which can be used to answer these and other questions. We are in the process of planning additional data analyses, for example comparing the MDL with government funding, analyzing donor differences by gender or geography, and more.
